# Development of control of attention from different perspectives

**DOI:** 10.3389/fpsyg.2014.01000

**Published:** 2014-09-08

**Authors:** Nicole Wetzel

**Affiliations:** Cognitive incl. Biological Psychology, Institute of Psychology, University of LeipzigLeipzig, Germany

**Keywords:** development, attention control, distraction, task-irrelevant, children, involuntary attention

The present Research Topic “It's irrelevant for the task but interesting!—How children process and attend to task-irrelevant information” comprises two aspects of cognitive control from a developmental point of view. The first aspect includes the development of children's attention control in different contexts such as emotion processing or novelty processing. The second aspect focuses on the development of children's ability to inhibit actions. The Research Topic addresses research with healthy children and with children suffering from the Attention Deficit Hyperactivity Disorder (ADHD). It brings together different perspectives on the development of attention control during infancy, childhood, and adolescence. This combination can contribute to new perspectives for future research and can enhance our knowledge about the development of cognitive functions, particularly about the developmental pathway of mechanisms of attention control.

## The development of attention control

The information processing capacity of our brain is limited and we cannot process all incoming stimuli concurrently—we need to select. The mechanisms of *selective attention* facilitate processing of goal-relevant events and inhibit the processing of goal-irrelevant events. Selective attention is a key function of the human information processing system and is essential for most other cognitive functions like learning, thinking, or memory.

The control of attention develops until the second decade of life and is closely related to the maturation of the brain, particularly of the prefrontal cortex (e.g., Fuster, 2002). With increasing maturation of the prefrontal cortex children's behavior becomes more controlled (Fuster, 2002) and working memory abilities increase (e.g., Gathercole et al., 2004; Luna et al., 2004). This results in more available resources for maintaining task-relevant processes. During this phase the brain's ability to shield against distraction of attention increases (e.g., Olesen et al., 2006; Wetzel et al., 2006; Wetzel and Schröger, 2014).

In the following section a short overview about the studies included in the Research Topic is given. The overview is arranged by the topic and in an alphabetical order of authors' surname within topics.

## Development of involuntary attention and distraction

Háden et al. (2013) performed two event-related potential (ERP) studies with 1–3-day-old newborns. They examined whether artificial noise segments and environmental novel sounds are processed in a context-dependent manner. The authors conclude from the results that neonates process environmental novel sounds but not noise sounds together with their context. The authors point out that the prerequisites of context-based perceptual object formation are present already at birth.

Klatte et al. (2013) provide a detailed review on environmental noise effects on cognitive performance in children. The authors give an overview of studies investigating children's acute or chronic exposure to noise and its impact on speech perception, listening comprehension, or reading performance. The authors emphasize that children are more susceptible to noise exposure than adults. Furthermore, children suffering from language or attention disorder, or second-language learners are more impaired by noise than controls.

Kushnerenko et al. (2013) provide a detailed review on auditory involuntary attention processes in the context of unexpected sounds within the first year of life. They particularly focus on the developmental pathway of the processing of genuine novel distractor sounds and repeatedly presented high-energy deviant sounds. The authors suggest explanations for age-related changes in the underlying brain activity and discuss their hypothesis also in the context of clinical research. Moreover, they discuss parallels of the infant ERP pattern to those of adults and point to methodological issues regarding ERPs and specific problems in infants.

Ruhnau et al. (2013) examined the involuntary change detection, orienting of attention, and behavioral distraction during an auditory-visual oddball task. They measured electro- and magnetoencephalographic brain activity in 9–10-year-old children and adults. The authors observed similar behavioral distraction effects and an early and a late distractor-related mismatch response located at auditory cortex areas in children and adults. Children's brain responses were delayed or showed different distribution over the scalp. The authors conclude that not all neurophysiological aspects of the underlying mechanisms are mature in late childhood.

Sussman (2013) investigated the modulation of neural activity by selective attention processes in adolescents. She analyzed distractor-related brain activity associated with change detection mechanisms (mismatch negativity) as a function of the task. In three conditions she presented the same sequence of sounds but asked adolescents to attend to different features of the stimulation or to ignore sounds. The author concludes from ERP results that selective attention modifies neural activity associated with change detection to support performance goals in adolescents.

## The impact of emotion on attention control and response inhibition

Heim et al. (2013) investigated the impact of affective stimuli embedded in rapid visual streams on attention capture in early adolescence. They utilized a rapid serial visual presentation paradigm and measured accuracy of target detection. The authors conclude from results that processing of affective stimuli is prioritized and interferes with subsequent target processing during the critical attentional blink interval. Age-related differences in the relation between distraction effects and the limitation of the available capacity are discussed in the framework of the use of alternative cognitive strategies.

Schel and Crone (2013) examined the influence of contextual relevant and irrelevant emotions on response inhibition. They presented a go/nogo task to a large age range (6–25 years). The authors report a linearly increasing response inhibition performance with age. Task-relevant emotions affect performance stronger than task-irrelevant emotions, particularly in young children. The lower effect of irrelevant emotions on response inhibition is supported by a free choice condition. Furthermore, the authors report enhanced performance in the context of relevant positive emotions compared to negative emotions, especially in young children.

## Distraction of attention in children suffering from ADHD

Children suffering from ADHD are frequently highly distractible by task-irrelevant events. Aboitiz et al. (2014) provide a detailed review about associated brain networks and their dysfunctions including pharmacologic, brain imaging, and electrophysiological studies. In that framework the authors discuss potential relations between catecholaminergic signaling networks and large-scale cortical networks regulating behavior.

## Conclusion

In sum, using different paradigms and stimuli and measuring different parameters of attention control indicate long lasting development of children's successful handling of task-irrelevant information. Finally, as also reflected in the Research Topic, the groups of toddlers and kindergarten children are underrepresented in this research (see also, Wetzel and Schröger, 2014). With a view to future research we need more systematic developmental and longitudinal studies investigating attention control over a wide age-range.

### Conflict of interest statement

The author declares that the research was conducted in the absence of any commercial or financial relationships that could be construed as a potential conflict of interest.
